# Supplementary material for: Disentangling plant- and environment-mediated drivers of active rhizosphere bacterial community dynamics during short-term drought
Source: Nat Commun. 2024 Jul 27;15:6347. doi: 10.1038/s41467-024-50463-1 (PMC11283566; doi:10.1038/s41467-024-50463-1)
Supplement: Supplementary file 1 — Supplementary Information [file 41467_2024_50463_MOESM1_ESM.pdf]

Supplementary Information for

Disentangling plant- and environment-mediated drivers of active rhizosphere bacterial community dynamics during short-term drought

Sreejata Bandopadhyay, Xingxing Li, Alan W. Bowsher, Robert L. Last, Ashley Shade

Methods: Accounting for phantom taxa and designating the active community members

After generation of OTU and taxonomy tables, all subsequent downstream processing were performed in R version 4.0. The R package decontam [1] revealed the library sizes for the negative controls and the true samples and helped to determine the number and identity of contaminants in the dataset (Supplementary Fig. **8A** and **8B**). This package was used to remove contaminants from the dataset by using the prevalence method. Contaminating OTUs, mitochondria, and chloroplast sequences were filtered from the datasets. Based on rarefaction curves, a subsampling depth of 15,000 reads was selected for both datasets (Supplementary **Fig. 8C**). After subsampling to equal depth of 15,000 reads, 16S rRNA to rRNA gene ratios (hereafter, 16S rRNA:rRNA gene) were computed from the DNA and cDNA datasets based on two methods as described in [2]; specifically method 1 and method 2 therein. Briefly, these methods differed in their level of conservativeness when accounting for OTUs that have sequences detected in the cDNA (suggesting active transcription at the time of sampling), but no detected sequences in the DNA. Thus, these taxa were potentially in low abundance so that the DNA sequencing was not sensitive enough to detect them, but their transcripts were in relatively higher abundance and could be readily detected given the sequencing depth. Method 1 sets the ratio for all OTUs with cDNA>0 and DNA=0 to 100, and method 2 computes the ratio for all OTUs with cDNA > 0 and DNA= 0 by setting the DNA=1. Both methods produced similar results for all ecological statistics when active taxa were designated at a ratio threshold >=1. When ratio threshold was set to >1, method 1 overestimated the phantom OTUs remaining compared to method 2. However, we proceeded with ratio threshold >=1 so we could account for all those taxa which were present in equal abundances in DNA and cDNA datasets, and hence contributed to a ratio=1. Given that similar results were observed at this threshold for both methods, we picked method 2 to account for phantom taxa. Overall trends in beta diversity did not change, whether singletons were removed or kept, or whether the phantom taxa were included or excluded. In general, most of the phantom OTUs in the rarefied dataset (>10,000 out of 14,479 phantom taxa; total OTUs in rarefied dataset was 48,647) had very low occupancy across all the 212 samples in the cDNA dataset. We define phantom taxa here as those OTUs

whose DNA counts (row sums) across samples is equal to zero, but cDNA counts (row sums) are greater than zero. For around 8000 phantom OTUs, around 100% of all instances of detection in the cDNA dataset across 212 samples had cDNA read equal to 1. Thus, for the purpose of analyzing the active community data, we kept the singletons and filtered to only those phantom taxa that were detected in at least 5% of the samples in the cDNA dataset. Around 922 taxa out of the 14,479 phantom taxa (6.4%) in the rarefied dataset were detected in the DNA reads of the unrarefied dataset, indicating that rarefaction could have led to the loss of DNA detected in the final dataset, though that percentage is very small. For those phantom taxa detected in the unrarefied dataset, the distribution of DNA counts revealed that most of those taxa were rare with reads summed across samples mostly adding up to less than 20 reads. Not surprisingly, only 0.04% of the total DNA reads in the unrarefied dataset were attributed to those 922 phantom taxa. The occupancy of most of those 922 phantom taxa in the unrarefied DNA dataset were also less than 1%. After selecting phantom taxa based on the threshold value of 5% occupancy across cDNA samples, 214 phantom OTUs remained out of the 14,479 taxa. A total of 12,388 OTUs remained after filtering by 16S rRNA:rRNA gene ratio threshold >=1 and selecting phantom taxa at 5% detection. The abundances of these OTUs were filtered to the DNA counts (for phantom taxa this was set to 1) and all analyses were performed based on this final active dataset filtered to DNA abundance. Consequently, while every sample was initially rarefied to 15,000 reads, each sample's active community varied slightly in total reads.

Methods: Selection of active OTUs

The percent phantom taxa in the rarefied dataset were assessed, i.e., those taxa which have RNA reads but lack DNA reads. Around 30-50 % of all OTUs were phantom taxa in the bean and switchgrass dataset. Roughly 10% of all OTUs were singleton phantom taxa in both switchgrass and bean dataset.

We analyzed the percent active OTUs in the dataset after using the two methods for ratio calculation; method 1: setting all cDNA>0 and DNA=0 to ratio 100 and method 2: setting all DNA= 0 to DNA=1. The percent active OTUs maintained the same overall trend across sampling days for both bean and switchgrass planted and unplanted treatments irrespective of method and ratio threshold (>1 versus >=1) with slight decreases in active OTUs when threshold was > 1 compared >=1 (associated code to reproduce results is included).

Results: Metabolome analysis of switchgrass

To unravel the global differences between the metabolomes of switchgrass rhizosphere and bulk soil before and after the drought treatment, we carried out untargeted LC-MS based metabolomics analysis of

soils collected during the drought-treatment experiment. In addition, we also profiled the global changes that occurred in metabolomes of switchgrass shoot and root caused by the drought treatment (Supplementary **Fig. 7**). The metabolite profiling revealed a total of 3,532 distinct metabolite features whose maximum abundance among the biological samples was ≥ 500 (**Data 1 - 3, Supplementary Data File**). A comparative analysis revealed 1051 root-, 538 shoot- and 231 soil- specific features (Supplementary **Fig. 1A**). Root and shoot shared 1551, root and soil shared 883 and leaf and soil shared 750 features, while 736 features were shared by all three groups (Supplementary **Fig. 1A**). The normalized abundance of features for soil were used for principal component analysis (PCA), which revealed the drought and watered planted (rhizosphere) soil separating from the rest soil types, including all the unplanted (bulk) soil and planted soil on the day 0 (the "Pre-drought" condition, Supplementary **Fig. 1B**). These metabolite differences indicate that the soil metabolites were altered by planting the switchgrass after five days. To further explore the changes occurring in the metabolome of drought-treated planted soil, a scatter plot based on PCA scores was used. The result showed some divergence between the drought and watered planted soil samples (Supplementary **Fig. 1C**), but this was not statistically supported. A variable of importance (VIP) coefficient was generated from the PLS-DA model for each metabolite feature. The top 15 most differentially accumulated features (with the highest VIP coefficient) between the different types of soil are shown in **Figure 3**. The feature, *10.11_1000.5271n*, has the highest VIP coefficient indicating it as the most important variable among altered metabolites for separation of the metabolomes (**Figure 3**). The LC-MS/MS spectra revealed this metabolite feature is a previously identified switchgrass mono-glycosylated steroidal saponin [3] (Supplementary **Fig. 2)**. When the top 50 features with the highest VIP coefficients were used to generate a heatmap with hierarchical clustering, the planted and unplanted soil samples formed two distinct clusters that separated from the other soil samples including the pre-drought planted and unplanted soil (Supplementary **Fig. 1D**). Among these important features, a few were annotated as specialized metabolites, including a few previously reported switchgrass saponins [3] and root accumulating diterpenoids with elevated concentrations in the drought treated planted soil samples [3,4] (**Data 4, Supplementary Data File**). The higher abundances for these specialized metabolites in the drought planted soil implied that switchgrass might release them into the rhizosphere soil carrying out certain biological functions when stressed by drought.

Supplementary Table 1. Differences in gravimetric soil moisture and shoot biomass by experimental factor as assessed by a three-way ANOVA with interactions (Type III). The F-statistics and p values are reported with significance indicated at p<=0.05*, p<=0.01**, and p<=0.001***. Source data are provided as a Source Data file.

Factor	Gravimetric soil moisture				Shoot biomass			
	Bean (n=100, without pre-drought samples)		Switchgrass (n=100, without pre-drought samples)		Bean (n=50, without pre-drought samples)		Switchgrass (n=50, without pre-drought samples)	
	F value	p value	F value	p value	F value	p value	F value	p value
planted	1160.21 ***	<0.001	6.66 *	0.01				
drought	165.49 ***	<0.001	28.43 ***	<0.001	21.18 ***	<0.001	0.30	0.59
sampling day	7.52 ***	<0.001	0.20	0.94	1.89	0.13	3.43 *	0.02
planted:drought	31.46 ***	<0.001	0.00	0.98				
planted:sampling day	3.41 *	0.01	1.01	0.41				
drought:sampling day	11.97 ***	<0.001	0.24	0.91	1.49	0.22	1.32	0.28
planted:drought:sampling day	2.84 *	0.03	0.39	0.82				

Supplementary Table 2. Permuted analysis of variance (PERMANOVA) analysis to determine differences in microbiome structure given experimental treatments. Sums of squares, degrees of freedom, R-squared values, Pseudo F and p values are reported. The significance of each test is indicated at p<=0.05*, p<=0.01**, and p<=0.001***. Adjustments for multiple comparisons were not conducted due to the factors mentioned in Anderson [5] and Day and Quinn [6]. Source data are provided as a Source Data file.

Factor	Switchgrass (n=97, without pre-drought samples)					Bean (n=96, without pre-drought samples)				
	Sums of squares	Degrees of freedom (DF)	R^2	Pseudo-F	p value	Sums of squares	Degrees of freedom (DF)	R^2	Pseudo-F	p value
planted	0.56	1	0.03	3.30***	0.001	3.12	1	0.17	21.57***	0.001
drought	0.28	1	0.02	1.64***	0.001	0.39	1	0.02	2.71***	0.001
sampling day	0.87	4	0.05	1.28***	0.001	0.95	4	0.05	1.65***	0.001
planted:drought	0.19	1	0.01	1.13	0.18	0.27	1	0.01	1.84*	0.03
planted:sampling day	0.79	4	0.05	1.16*	0.04	0.75	4	0.04	1.29*	0.04
drought:sampling day	0.87	4	0.05	1.27**	0.002	0.78	4	0.04	1.35*	0.02
planted:drought:sampling day	0.74	4	0.04	1.08	0.12	0.68	4	0.04	1.17	0.12

Supplementary Table 3. Differences in the number of observed active OTUs (richness) across experimental factors, as assessed using a three-way ANOVA with interactions (Type III tests). F-statistics and p values are reported, and significance is indicated at p<=0.05*, p<=0.01**, and p<=0.001***. Source data are provided as a Source Data file.

Factor	Observed OTUs			
	Bean (n=96)		Switchgrass (n=97)	
	F value	p value	F value	p value
planted	60.12 ***	<0.001	0.35	0.56
drought	0.07	0.80	1.99	0.16
sampling day	1.61	0.18	0.79	0.53
planted:drought	2.32	0.13	4.30 *	0.04
planted:sampling day	2.12	0.09	0.49	0.74
drought:sampling day	1.23	0.31	1.16	0.34
planted:drought:sampling day	1.42	0.24	0.49	0.74

Supplementary Table 4. Chemical and physical analysis of field-collected soils for bean and switchgrass.

Soil Sample	Switchgrass (*Panicum virgatum*)	Bean (*Phaseolus vulgaris*)
Soil pH	6	6.7
Lime Index	69	71
Phosphorus (ppm)	21	200
Potassium (ppm)	72	109
Magnesium (ppm)	124	88
Calcium (ppm)	913	574
CEC (meq/100g)	7	3.9
K	3.2	7.2
Mg	17.9	18.9
Ca	78.9	73.9
Organic Matter (%)	2	1.1
Nitrate-N (ppm)	3.5	2.5
Ammonium-N (ppm)	1.6	0.6
Soil moisture (%)	14.1	13.7

Supplementary Table 5. Water addition schedule by treatment for switchgrass.

Treatment date (Year-2019)	Water added for watered treatment	Water added for drought treatment	Sampling day (sampling done without watering that morning)
Sat Apr 27	10 ml	5 ml	Pre-drought – Day 0
Sun Apr 28	0 ml	0 ml	
Mon Apr 29	10 ml	3 ml	Day 2
Tues Apr 30	0 ml	0 ml	Day 3
Wed May 1	10 ml	1 ml	Day 4
Thurs May 2	10 ml	0 ml	Day 5
Fri May 3	n/a	n/a	Day 6

Supplementary Table 6. Water addition schedule by treatment for bean.

Treatment Date (Year-2019)	Water added for watered treatments	Water added for drought treatments	Sampling day (sampling done without watering that morning)
Wed, May 22	15ml morning, 15ml afternoon.	8ml morning, 8ml afternoon	Pre-drought – Day 0
Thurs, May 23	15ml morning, 15ml afternoon.	6ml morning, 6ml afternoon	
Fri, May 24	15ml morning, 15ml afternoon.	4ml morning, 4ml afternoon	Day 2
Sat, May 25	15ml morning, 15ml afternoon.	2ml morning, 2ml afternoon	Day 3
Sun, May 26	15ml morning, 15ml afternoon.	0ml morning, 0ml afternoon	Day 4
Mon, May 27	15ml morning, 15ml afternoon.	0ml morning, 0ml afternoon	Day 5
Tues, May 28	n/a	n/a	Day 6

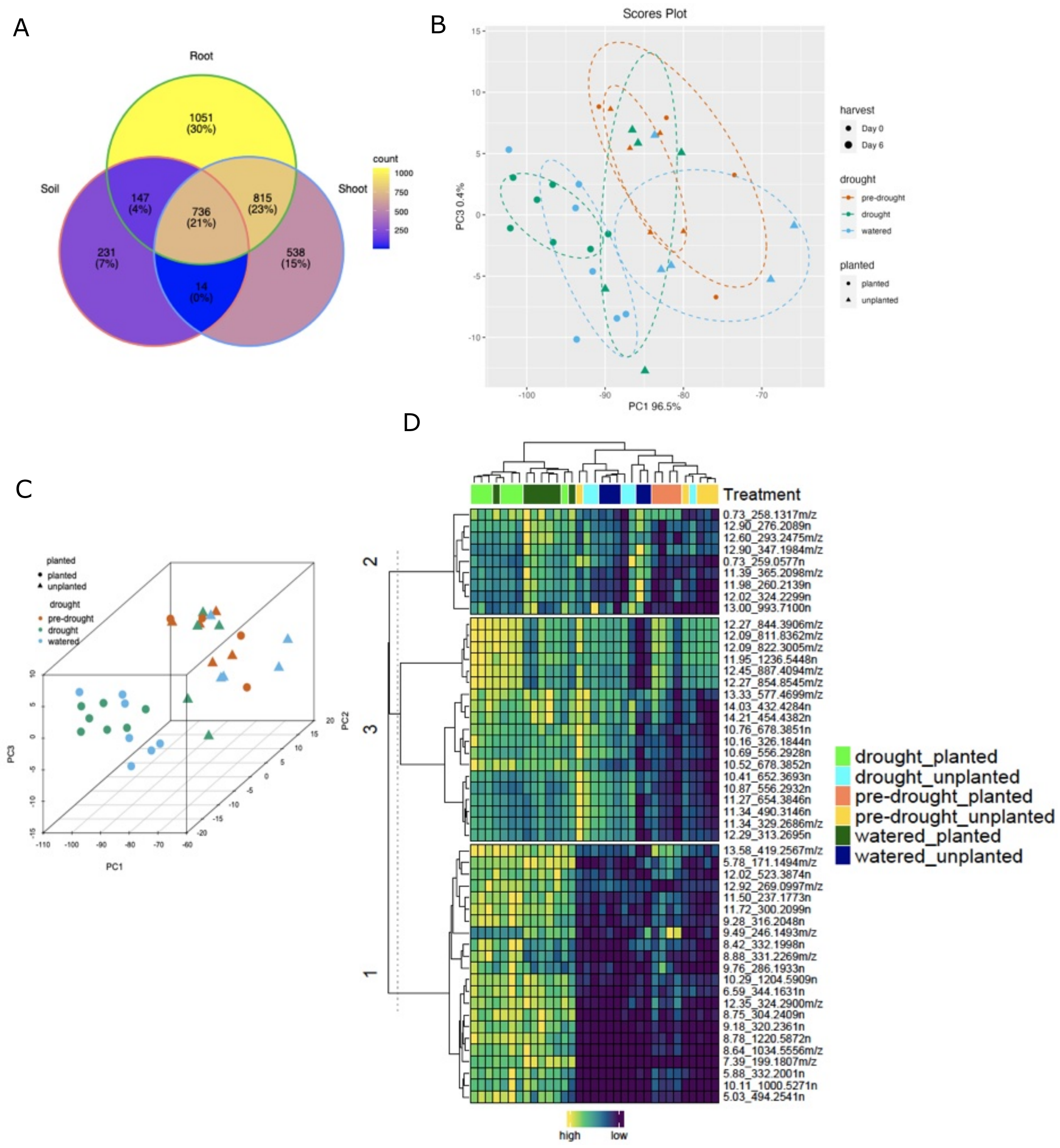

Supplementary Fig. 1. The untargeted metabolomics revealed metabolite differences between the soil types. A. Venn diagram documenting comparative metabolite profiles as well as number of specific and shared features identified in switchgrass root, shoot and soil. B. PCA scores plot of the metabolomes of different soil types showing the principal components PC1 and PC3. C. 3-D scatter plot of PCA scores plot of the metabolomes of different soil types showing PC1, 2 and 3. D. Heatmap showing relative abundances of the top 50 PLS-DA most important features (vertical axis) across the biological samples

(horizontal axis). For the hierarchical clustering of features, Euclidean and Ward.D were used as the distance measure and clustering method respectively (sample size root (n)=30, shoot (n)=30, soil(n)=49). Source data are provided as a Source Data file.

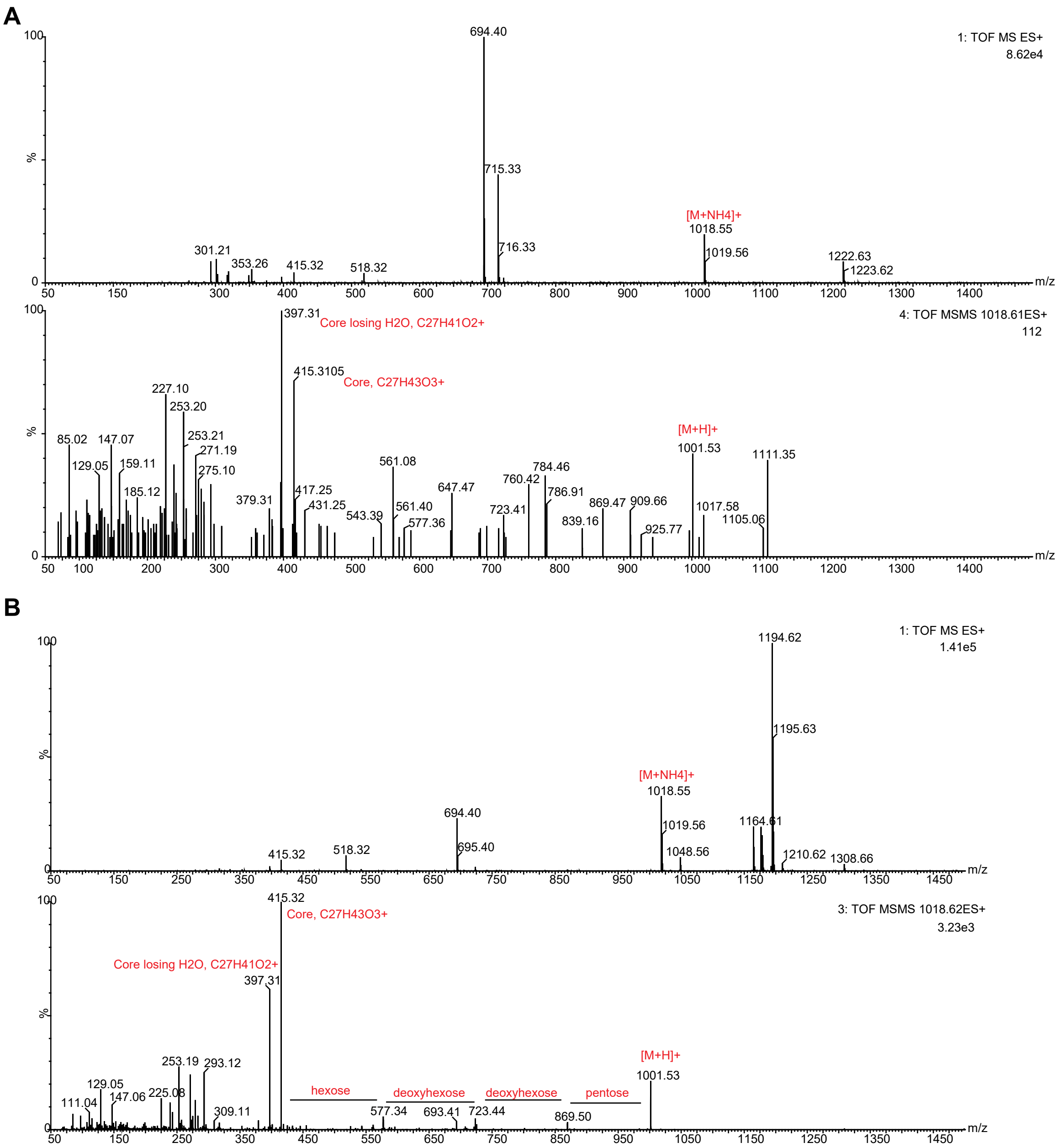

Supplementary Fig. 2. A. MS/MS spectra for the feature, *10.11_1000.5271n,* obtained by the positive LC-MS analysis, DDA mode. Top trace: survey scan; bottom trace: MS/MS. The molecular ions and sapogenin core fragment ions were annotated. M, molecular ion. **B.** MS/MS spectra for the feature detected in switchgrass roots in Li, et al. [3] that we speculate *10.11_1000.5271n* to be. Source data for Fig. 2. A. are provided as a Source Data file.

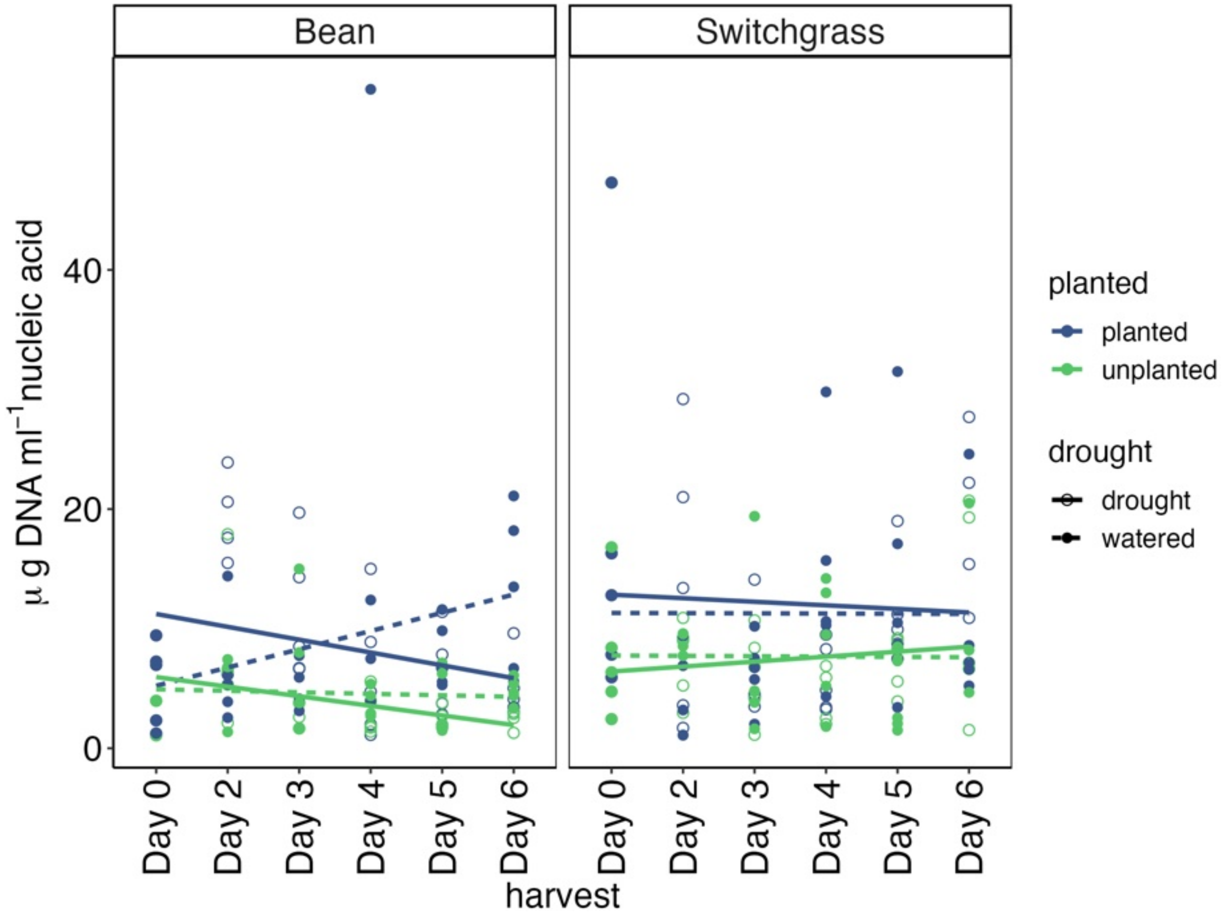

Supplementary Fig. 3. Recovered DNA concentrations of soil samples by crop, treatment, and time point. DNA was measured using Qubit 2.0 with the dsDNA BR assay kit. Sample size for bean (n)=102, sample size for switchgrass (n)=105. Source data are provided as a Source Data file.

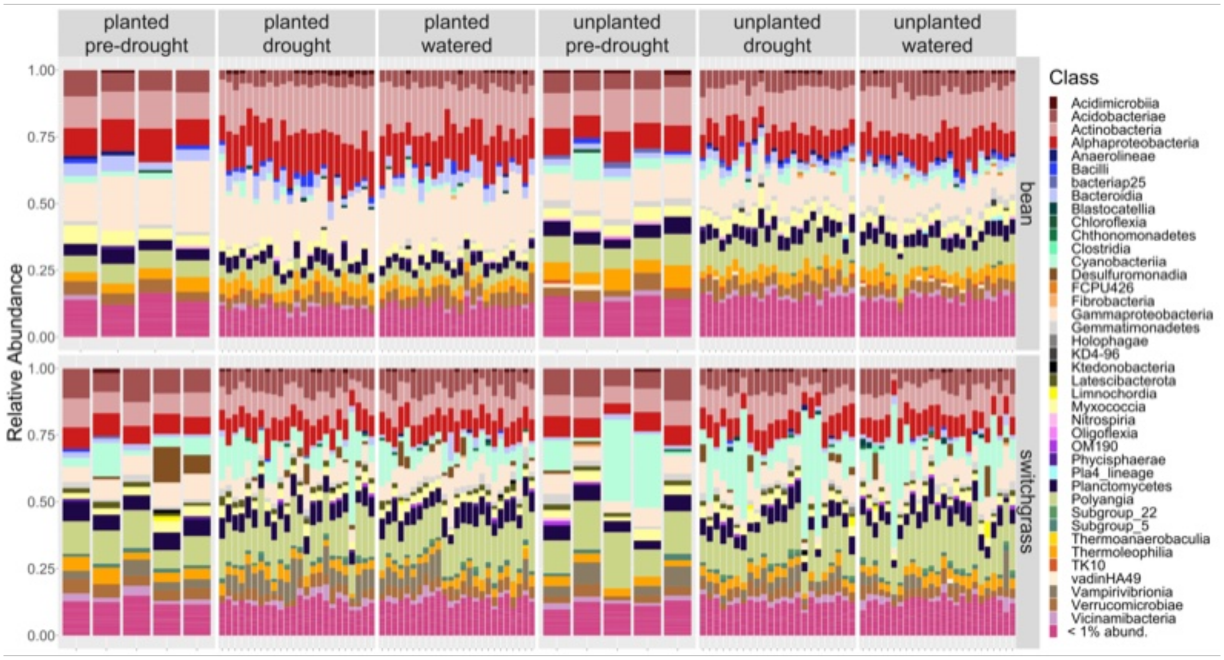

Supplementary Fig. 4. Stacked bar plots depicting the Class-level, active bacterial community composition by treatment for bean and switchgrass. All samples collected are shown (n=212). Source data are provided as a Source Data file.

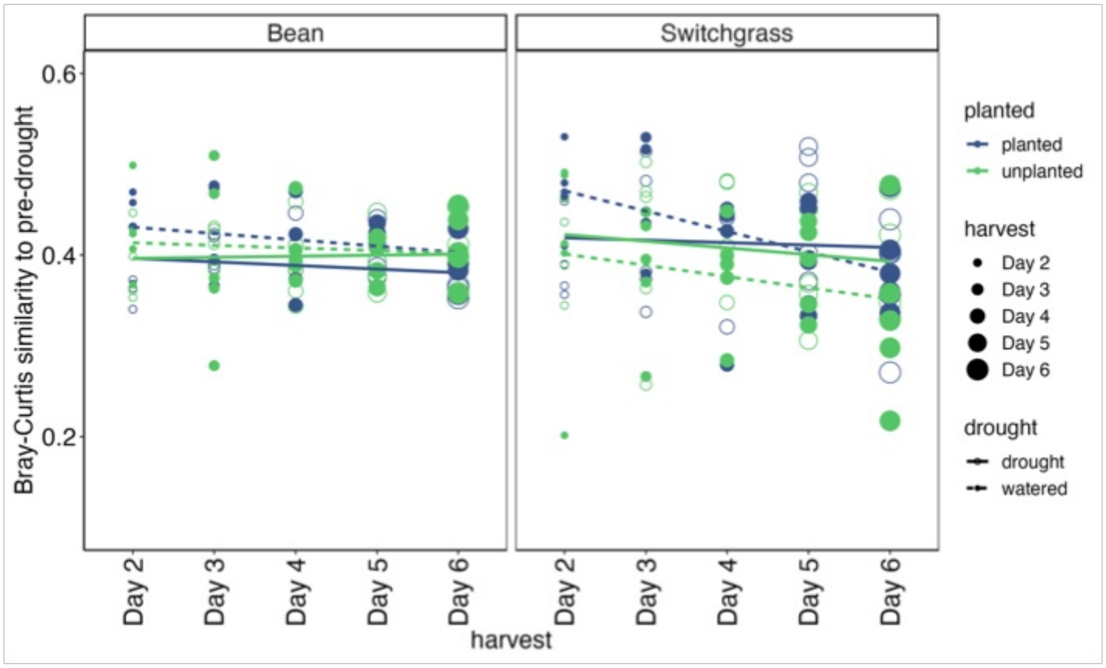

Supplementary Fig. 5. For each experimental treatment, changes in Bray Curtis similarity to the pre-drought community over time and/or with increasing severity. Sampling day is indicated by the symbol size, and treatment by colors (blue is planted, green is unplanted) and line type (solid is drought and dashed is watered). The linear regressions were insignificant for any treatment, except for the watered, planted switchgrass (blue, dashed line on the right panel). Sample size for bean (n)=87, sample size for switchgrass (n)=97. Source data are provided as a Source Data file.

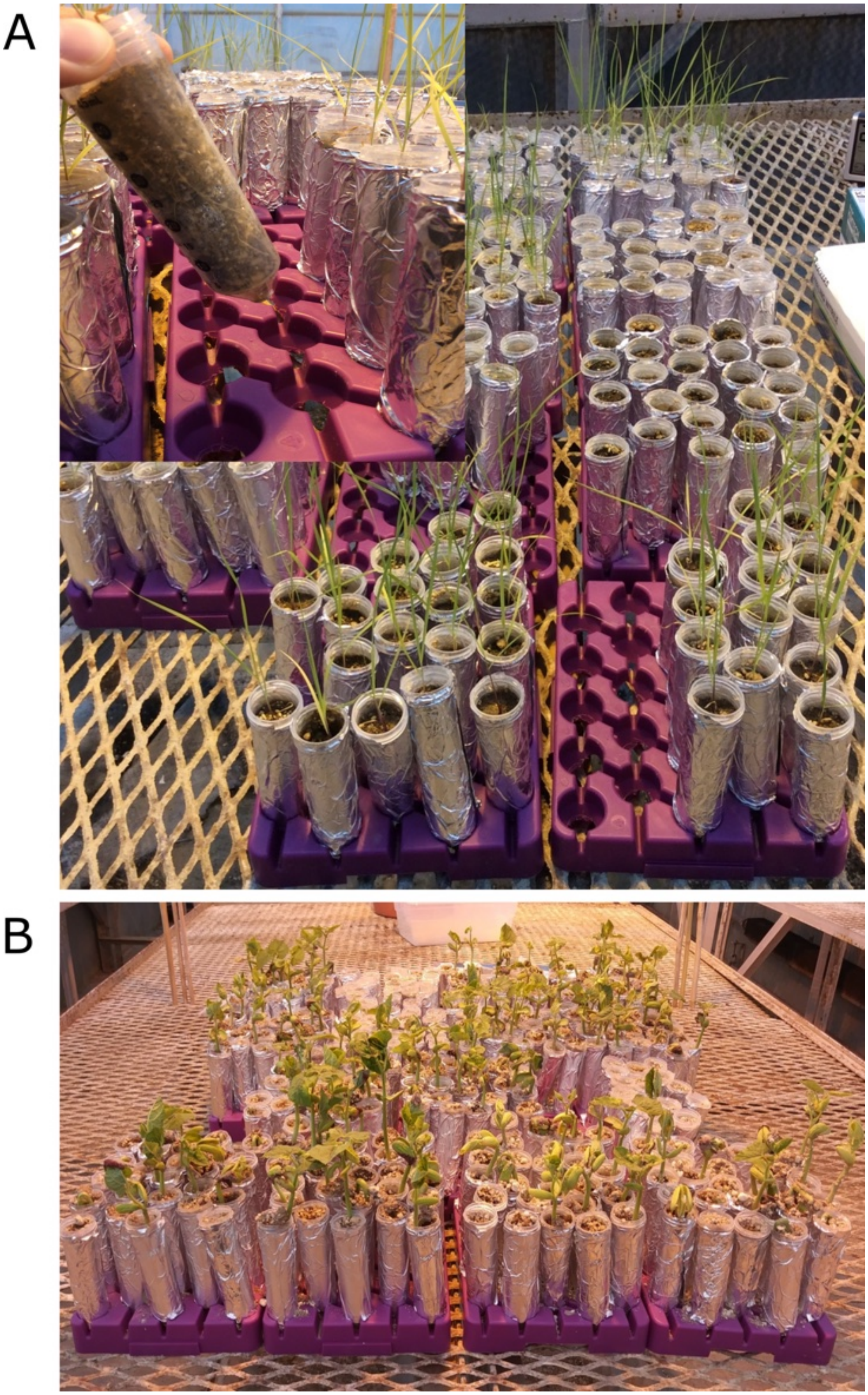

Supplementary Fig. 6. Photo of experimental set up in the greenhouse for A) switchgrass (inset- tube showing switchgrass roots grown to fill to the inside edge of the tube) and B) bean.

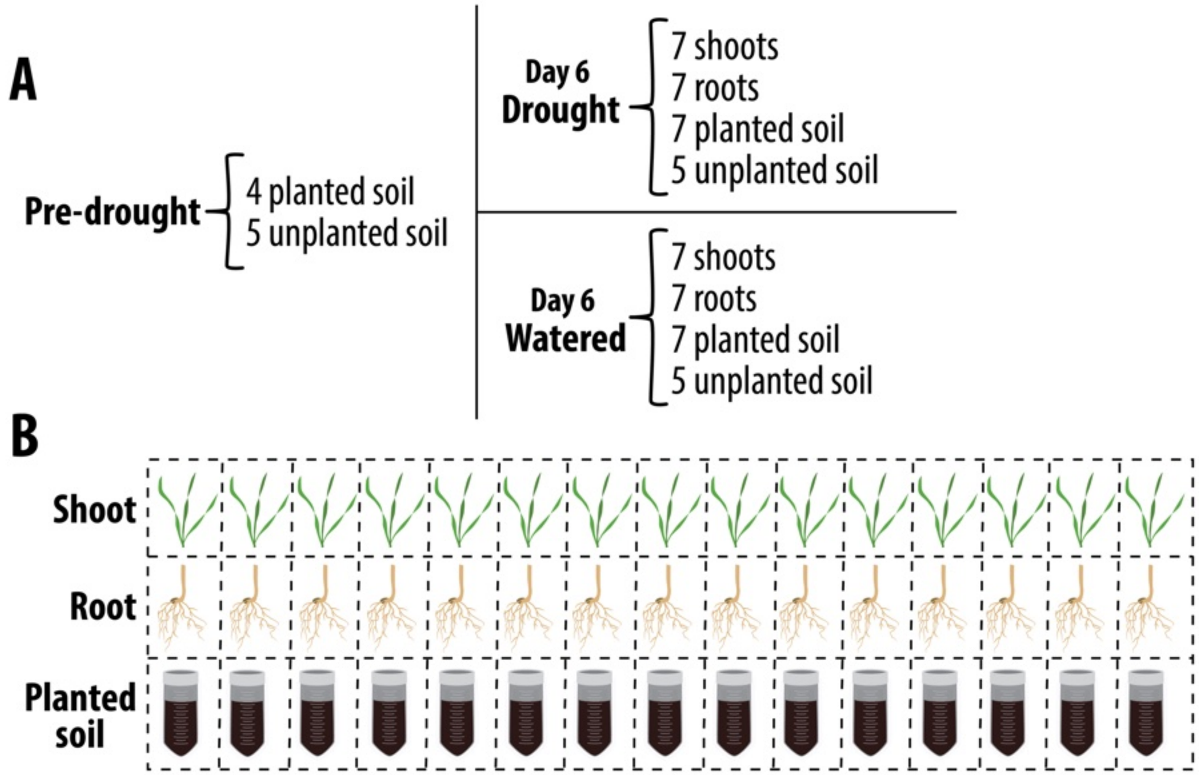

Supplementary Fig. 7. Sample collection for the switchgrass untargeted metabolomics. **A.** Sample panel with the numbers of replicates indicated. **B.** The drought and watered switchgrass tissue and planted soil replicates were combined from the samples that were collected from individual plants.

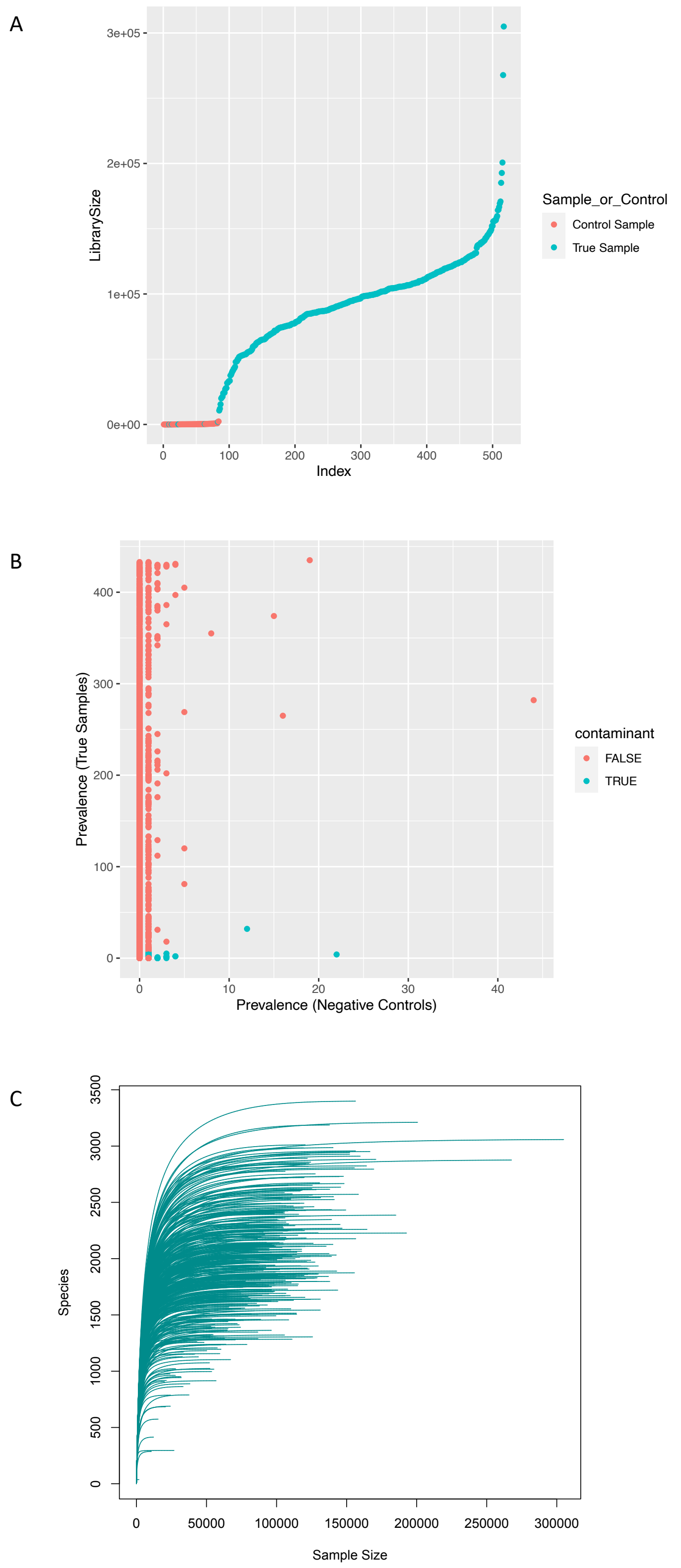
A
LibrarySize
Index
Sample_or_Control
Control Sample
True Sample
B
Prevalence (True Samples)
Prevalence (Negative Controls)
contaminant
FALSE
TRUE
C
Species
Sample Size

Supplementary Fig. 8. Sequencing quality control checks. (A) Library sizes of true samples (blue) and negative control (red) samples for both DNA and cDNA-sequenced samples. (B) OTUs designated as either true or false contaminants based on their prevalence in true samples and negative controls using package decontam in R. (C) Rarefaction curves for all DNA and cDNA samples (pre-rarefaction). Curves start to plateau out at 13-15k reads (n=438). Source data are provided as a Source Data file.

REFERENCES

1 Davis, N. M., Proctor, D. M., Holmes, S. P., Relman, D. A. & Callahan, B. J. Simple statistical identification and removal of contaminant sequences in marker-gene and metagenomics data. *Microbiome* **6**, 226, doi:10.1186/s40168-018-0605-2 (2018).

2 Bowsher, A. W., Kearns, P. J. & Shade, A. 16S rRNA/rRNA Gene Ratios and Cell Activity Staining Reveal Consistent Patterns of Microbial Activity in Plant-Associated Soil. *mSystems* **4**, doi:10.1128/mSystems.00003-19 (2019).

3 Li, X., Sarma, S. J., Sumner, L. W., Jones, A. D. & Last, R. L. Switchgrass Metabolomics Reveals Striking Genotypic and Developmental Differences in Specialized Metabolic Phenotypes. *J. Agric. Food Chem.* **70**, 8010-8023, doi:10.1021/acs.jafc.2c01306 (2022).

4 Tiedge, K. *et al.* Comparative transcriptomics and metabolomics reveal specialized metabolite drought stress responses in switchgrass (Panicum virgatum). *New Phytol.* **236**, 1393-1408, doi:https://doi.org/10.1111/nph.18443 (2022).

5 Anderson, M. PERMANOVA+ for PRIMER: guide to software and statistical methods. *Primer-E Limited.* (2008).

6 Day, R. & Quinn, G. Comparisons of treatments after an analysis of variance in ecology. *Ecological monographs* **59**, 433-463 (1989).
