# Supplementary material for: Disentangling plant- and environment-mediated drivers of active rhizosphere bacterial community dynamics during short-term drought
Source: Nat Commun. 2024 Jul 27;15:6347. doi: 10.1038/s41467-024-50463-1 (PMC11283566; doi:10.1038/s41467-024-50463-1)
Supplement: Supplementary file 3 — Description of additional supplementary files [file 41467_2024_50463_MOESM3_ESM.pdf]

Description of Additional Supplementary Files

Supplementary Data 1: The metabolite features identified from Cave-in-Rock shoot tissues (with abundance ≥ 500) by the positive mode LC-MS.

Supplementary Data 2 : The metabolite features identified from Cave-in-Rock root tissues (with abundance ≥ 500) by the positive mode LC-MS.

Supplementary Data 3: The metabolite features identified from all soil samples (with abundance ≥ 500) by the positive mode LC-MS analysis.

Supplementary Data 4: The top 50 PLS-DA important metabolite features (obtained by positive mode LC-MS analysis). Annotations were performed by searching the online mass spec databases through the Progenesis QI or using CANOPUS machine function build in the SIRIUS 4 (https://bio.informatik.uni-jena.de/sirius/) (Materials and Methods). The ones annotated by CANOPUS are in boldface. a Compounds were previously reported in the Tiedge et al. (2022); b Compounds were previously reported in the Li et al. (2022). c Identification level (A; B; C)- (A) standard or NMR; (B(i)) confident match based on MS/MS and (B(ii)) confident match using in-silico MS/MS approaches and (B(iii)) partial match based on MS/MS and (C(i)) confident match based on MSn and (C(ii)) confident match using in-silico MSn approaches and (C(iii)) partial match based on MSn; (D) MS only. These are based on the criteria for metabolite identification (Sumner et al., 2007).
