# Supplementary material for: Disentangling plant- and environment-mediated drivers of active rhizosphere bacterial community dynamics during short-term drought
Source: Nat Commun. 2024 Jul 27;15:6347. doi: 10.1038/s41467-024-50463-1 (PMC11283566; doi:10.1038/s41467-024-50463-1)
Supplement: Supplementary file 5 — Reporting Summary [file 41467_2024_50463_MOESM5_ESM.pdf]

nature portfolio

Corresponding author(s): Ashley Shade

Last updated by author(s): 02/july/2024

Reporting Summary

Nature Portfolio wishes to improve the reproducibility of the work that we publish. This form provides structure for consistency and transparency in reporting. For further information on Nature Portfolio policies, see our Editorial Policies and the Editorial Policy Checklist.

Statistics

For all statistical analyses, confirm that the following items are present in the figure legend, table legend, main text, or Methods section.

n/a	Confirmed	
☐	☒	The exact sample size (*n*) for each experimental group/condition, given as a discrete number and unit of measurement
☐	☒	A statement on whether measurements were taken from distinct samples or whether the same sample was measured repeatedly
☐	☒	The statistical test(s) used AND whether they are one- or two-sided<br>*Only common tests should be described solely by name; describe more complex techniques in the Methods section.*
☐	☒	A description of all covariates tested
☐	☒	A description of any assumptions or corrections, such as tests of normality and adjustment for multiple comparisons
☐	☒	A full description of the statistical parameters including central tendency (e.g. means) or other basic estimates (e.g. regression coefficient) AND variation (e.g. standard deviation) or associated estimates of uncertainty (e.g. confidence intervals)
☐	☒	For null hypothesis testing, the test statistic (e.g. *F*, *t*, *r*) with confidence intervals, effect sizes, degrees of freedom and *P* value noted<br>*Give P values as exact values whenever suitable.*
☒	☐	For Bayesian analysis, information on the choice of priors and Markov chain Monte Carlo settings
☐	☒	For hierarchical and complex designs, identification of the appropriate level for tests and full reporting of outcomes
☐	☒	Estimates of effect sizes (e.g. Cohen's *d*, Pearson's *r*), indicating how they were calculated

Our web collection on statistics for biologists contains articles on many of the points above.

Software and code

Policy information about availability of computer code

Data collection	No software was used in data collection
Data analysis	Custom code was used for data analysis using R version 4.0. Code for data analysis used in this study can be found on GitHub at https://github.com/ShadeLab/PAPER_DroughtRhizobiome_Bandopadhyay_2023.<br>R packages used are mentioned in the manuscript. Briefly we used these R packages- phyloseq (version 1.38.0), tidyverse (version 1.3.2), ggplot2 (version 3.4.0), ggpubr (version 0.5.0), ggh4x (version 0.2.3.9000), grid (version 4.1.2), reshape2 (version 1.4.4), decontam (version 1.14), vegan (version 2.6-4), scales (version 1.2.1), multcompView (version 0.1-8), car (version 3.1-1), lsmeans (version 2.30-0), viridis (version 0.6.2), ape (version 5.6-2), pairwiseAdonis (version 0.4), usedist (version 0.4.0), dendextend (version 1.16.0), ggtree (version 3.2.1), emmeans (version 1.8.4-1)

For manuscripts utilizing custom algorithms or software that are central to the research but not yet described in published literature, software must be made available to editors and reviewers. We strongly encourage code deposition in a community repository (e.g. GitHub). See the Nature Portfolio guidelines for submitting code & software for further information.

Data

Policy information about availability of data

All manuscripts must include a data availability statement. This statement should provide the following information, where applicable:

- Accession codes, unique identifiers, or web links for publicly available datasets
- A description of any restrictions on data availability
- For clinical datasets or third party data, please ensure that the statement adheres to our policy

Sequence data for this study has been deposited to NCBI Sequence Read Archive under project number PRJNA862978.
The LC-MS metabolomics datasets are now publicly available on Dryad - 'Metabolomics data for Disentangling plant- and environment-mediated drivers of active rhizosphere bacterial community dynamics during short-term drought'. It has been assigned a digital object identifier (DOI): doi:10.5061/dryad.6t1g1jx5z. Access to the data files can be obtained using this URL: https://datadryad.org/stash/share/vkqIFDwKkWd0_U36mKWee1z1-pb3Xha3ajrZ7x_HO2c.
All raw data files used to generate and replicate figures throughout the manuscript have also been uploaded to Figshare and can be accessed here https://figshare.com/s/f5e432de0a1c930e85fd.

Research involving human participants, their data, or biological material

Policy information about studies with human participants or human data. See also policy information about sex, gender (identity/presentation), and sexual orientation and race, ethnicity and racism.

Reporting on sex and gender	NA
Reporting on race, ethnicity, or other socially relevant groupings	NA
Population characteristics	NA
Recruitment	NA
Ethics oversight	NA

Note that full information on the approval of the study protocol must also be provided in the manuscript.

Field-specific reporting

Please select the one below that is the best fit for your research. If you are not sure, read the appropriate sections before making your selection.

☐ Life sciences ☐ Behavioural & social sciences ☒ Ecological, evolutionary & environmental sciences

For a reference copy of the document with all sections, see nature.com/documents/nr-reporting-summary-flat.pdf

Ecological, evolutionary & environmental sciences study design

All studies must disclose on these points even when the disclosure is negative.

Study description	The study included drought and watered treatments applied to field collected rhizosphere soil samples in a greenhouse setting. Each treatment included 25 planted and 25 unplanted treatments totaling a 100 samples for bean and a 100 samples for switchgrass. Five replicate samples were collected at each sampling point. Treatment factors were crop (bean, switchgrass), drought (drought, watered), sampling day (day 0, 2, 3, 4, 5, 6) and planted levels (planted, unplanted). Effect of main factors and interactions are both tested in statistical tests performed.
Research sample	There was no specific research sample used in this study. We used field collected rhizosphere soil and used them to grow bean and switchgrass in the greenhouse drought experiment. We did this to include soil and plant legacy effects in the response of the plant and soil to the drought treatment.
Sampling strategy	For bean plants, sampling started on May 22, 2019 and ended on May 28, 2019 with data collection occurring on Day 0 (pre-drought), and on Days 2, 3, 4, 5 and 6 post drought initiation. This incremental time correspond to increased drought severity. Sampling for switchgrass was done in a separate experiment and started on April 27, 2019 and ended on May 3, 2019. Similar to bean plants, data collection occurred on Day 0 (pre-drought), and Days 2, 3, 4, 5 and 6 post drought initiation and corresponded to increased drought severity. We decided to chose this timeline based on pilot experiments done before the main experiment to ensure that drought severity treatment is effective and plants are viable during the experiment. Samples were collected from drought (planted, unplanted) and watered (planted, unplanted) treatments. An additional set of drought and watered plants were leveraged using the same timeline as described above to collect root, shoot and rhizosphere soil for metabolomics. We selected our sample size based on previous studies that show that 3-5 replications provide sufficient power to detect the effect of drought treatment on soil samples. For this study, we report all statistics based on 5 replicate samples per treatment.

Data collection	Sample and data were collected by leveraging a greenhouse experiment to understand the effect of short-tern drought on common bean and switchgrass rhizosphere microbiome dynamics and metabolomics. Well watered plants were grown and baseline rhizosphere soil samples collected before initiating treatments. Rhizosphere soil from both planted and unplanted treatments were collected at baseline (pre-drought) for microbiome analysis. Subsquently, a short-term drought of 6 days was initiated for both crops (bean and switchgrass). Plants were destructively harvested at 2, 3, 4, 5 and 6th day of drought wherein rhizosphere soil, plant roots and shoots were harvested for microbiome analysis, metabolomics, shoot biomass measurements and percent soil moisture.
Timing and spatial scale	All data were collected in the same greenhouse using field soil growing the respective plant. For bean plants, data collection started on May 22, 2019 and ended on May 28, 2019 with data collection occurring on Day 0 (pre-drought), and on Days 2, 3, 4, 5 and 6 post drought initiation. This incremental time correspond to increased drought severity. Data collection for switchgrass was done in a separate experiment and started on April 27, 2019 and ended on May 3, 2019. Similar to bean plants, data collection occurred on Day 0 (pre-drought), and Days 2, 3, 4, 5 and 6 post drought initiation and corresponded to increased drought severity. We decided to chose this timeline based on pilot experiments done before the main experiment to ensure drought severity treatment is effective and plants are viable during the experiment. Samples were collected from drought (planted, unplanted) and watered (planted, unplanted) treatments.
Data exclusions	For microbial community, data excluded from the analysis include those that did not pass rarefaction threshold and for which sequencing efforts were unsuccessful. DNA yield data does not include samples for which DNA could not be quantified via a Qubit fluorometer.
Reproducibility	Pilot tests were conducted to confirm accurate watering levels for each crop such that the drought treatment would be effective and plants would be viable. After standardizing the watering regime for well-watered and drought plants, the final experiment was performed which successfully reproduced the findings from the pilot studies. At least one pilot test was done in the greenhouse for both bean and switchgrass. Even though no statistical method was used to predetermine sample size, we opted for 5 replicates as they accurately captured any differences observed in plant physiology during the experiment.
Randomization	Allocation of planted and unplanted samples to either the watered or drought treatments was randomly done.
Blinding	Blinding was not relevant to our study because it is not human subjects research.

Did the study involve field work? ☒ Yes ☐ No

Field work, collection and transport

Field conditions	While temperature and rainfall were not noted during sampling, initial soil properties are noted in the manuscript Supplementary Information.
Location	Bean soil was collected from Montcalm Research Center on September 6, 2018, at Stanton, MI (43.350885, -85.177044), and had been recently planted with common bean (Phaseolus vulgaris var. Red Hawk) that year. Switchgrass samples were collected from the Great Lakes Bioenergy Research Center switchgrass plots at Lux Arbor Reserve (42.475224, -85.444979) in Denton, MI, on August 27, 2018. This site has been under continuous switchgrass (Panicum virgatum var. Cave-in-rock) cultivation since 2011.
Access & import/export	The switchgrass soil collections were planned with the Project Manager at Kellogg Biological Station in Hickory Corners, MI. A Site Use Request Form (SURF) was submitted and approved by the project manager before sampling. No specific permits for needed for the bean soil collection.
Disturbance	No disturbance was caused due to soil sampling.

Reporting for specific materials, systems and methods

We require information from authors about some types of materials, experimental systems and methods used in many studies. Here, indicate whether each material, system or method listed is relevant to your study. If you are not sure if a list item applies to your research, read the appropriate section before selecting a response.

Materials & experimental systems

n/a	Involved in the study	
☒	☐	Antibodies
☒	☐	Eukaryotic cell lines
☒	☐	Palaeontology and archaeology
☒	☐	Animals and other organisms
☒	☐	Clinical data
☒	☐	Dual use research of concern
☐	☒	Plants

Methods

n/a	Involved in the study	
☒	☐	ChIP-seq
☒	☐	Flow cytometry
☒	☐	MRI-based neuroimaging

Dual use research of concern

Policy information about dual use research of concern

Hazards

Could the accidental, deliberate or reckless misuse of agents or technologies generated in the work, or the application of information presented in the manuscript, pose a threat to:

No	Yes	
☒	☐	Public health
☒	☐	National security
☒	☐	Crops and/or livestock
☒	☐	Ecosystems
☒	☐	Any other significant area

Experiments of concern

Does the work involve any of these experiments of concern:

No	Yes	
☒	☐	Demonstrate how to render a vaccine ineffective
☒	☐	Confer resistance to therapeutically useful antibiotics or antiviral agents
☒	☐	Enhance the virulence of a pathogen or render a nonpathogen virulent
☒	☐	Increase transmissibility of a pathogen
☒	☐	Alter the host range of a pathogen
☒	☐	Enable evasion of diagnostic/detection modalities
☒	☐	Enable the weaponization of a biological agent or toxin
☒	☐	Any other potentially harmful combination of experiments and agents

Plants

Seed stocks	Switchgrass seeds were obtained from Sharp Bros. Seed of Mo., Inc and bean seeds were obtained from the Dry Bean Breeding and Genetics program at Michigan State University.
Novel plant genotypes	No novel plant genotypes were produced
Authentication	NA
